# Retention of Phytochemical Compounds and Antioxidative Activity in Traditional Baked Dish “proja” Made from Pigmented Maize

**DOI:** 10.3390/foods13172799

**Published:** 2024-09-03

**Authors:** Olivera Šimurina, Bojana Filipčev, Biljana Kiprovski, Zvonko Nježić, Elizabet Janić Hajnal, Ivica Đalović

**Affiliations:** 1 Institute of Food Technology, University of Novi Sad, Bul. cara Lazara, 21000 Novi Sad, Serbia; olivera.simurina@fins.uns.ac.rs (O.Š.);; 2 Institute of Field and Vegetable Crops, Maksima Gorkog 30, 21101 Novi Sad, Serbia; biljana.kiprovski@ifvcns.ns.ac.rs (B.K.); ivica.djalovic@ifvcns.ns.ac.rs (I.Đ.)

**Keywords:** phenolics, anthocyanins, flavonoids, inhibitory activity, thermal processing, phenolic compounds stability

## Abstract

Two genotypes of pigmented maize (black (BM) and red (RM)) were used as flour ingredients in several formulations of the traditional baked maize dish “proja”. This study investigated the stability of phytochemical compounds and antioxidant activity in proja as affected by baking and different acidity degrees of dough formulations. Compared to RM proja, all BM proja formulations were significantly higher in antioxidant compounds and exhibited the highest inhibitory activity (73–85%) against DPPH. There was a strong significant correlation between DPPH inhibition and total phenolics (r^2^ = 0.95), flavonoids (r^2^ = 0.96), and anthocyanins (r^2^ = 0.97) in baked proja. After baking, 67–85% of total phenolics were retained. The fate of flavonoids and anthocyanins after baking was variable: from 70% degradation to liberation. Dough acidity significantly and positively affected the content of phenolics, flavonoids, and anthocyanins in BM proja (r^2^ = 0.70, 0.82, and 0.47, respectively). Baking increased antioxidant activity against DPPH, ^•^OH, and O_2_^•−^ radicals in proja, except for ≈10% decline of DPPH inhibition in BM proja. In RM proja, retention of inhibitory capacity against O_2_^•−^ was highly correlated to flavonoid retention (r^2^ = 0.71). Using pigmented maize flour in proja baking resulted in proja with appreciable content of total phenolics, flavonoids, anthocyanins, and high antioxidant activity, confirming the significant improvement of the nutrient profile of this traditional food.

## 1. Introduction

In the last decade, pigmented maize genotypes have been extensively studied for potential benefits beyond their nutritional value, which have been associated with the considerable content of phytochemicals [1,2]. Like traditional yellow maize, pigmented maize is primarily a source of carbohydrates, mostly starch (76–84%) [3]. Later, the nutraceutical potential of coloured maize has been primarily attributed to the presence of pigments (anthocyanins), which exert high antioxidant activity. The dominant anthocyanins in pigmented maize are peonidin, cyanidin, pelargonidin, and malvidinin in the form of several glucoside derivates (reviewed in [3,4]). Besides anthocyanins, pigmented maize contains other phenolic compounds like phenolic acids and flavonoids, which also contribute to the health-promoting potential of coloured maize. Phenolic compounds in maize are predominantly located in the outermost layers of the grains and can be found in soluble-free, soluble-ester, and insoluble-bound forms [5]. The health effects of these compounds are influenced by their form and distribution within the kernel. Free phenolics are quickly absorbed and conjugated in the small intestine post-ingestion, resulting in lower levels of aglycones in the bloodstream. In contrast, insoluble phenolics are bound by covalent or non-covalent interactions with dietary fibres, which modulate their release from the food matrix: fibres can decrease the polyphenol absorption in the small intestine but increase their amount and release in the colon through colonic fermentation, thus exerting health-beneficial action [6]. The primary phenolic compounds in maize are phenolic acids, although phenolic amines and flavonoids have also been identified [7]. In coloured maize grains, significant variations in the levels of free and bound phenolics have been observed, likely due to differences in biological material and extraction techniques. Data generally indicate a predominance of insoluble phenolics, which are primarily bound to polysaccharides within the cell wall or linked to lignin. Free phenolics can be conjugated to simple sugars and amines [7]. Anthocyanins belong to the class of water-soluble flavonoids and are located in the pericarp and aleurone layers of the grains. The content and profile of anthocyanin compounds greatly vary among the pigmented maize genotypes; anthocyanin amounts are in ascending order from pink, blue, purple, and black maize [8].

Though it has been traditionally used as food in countries of Latin America, compared to yellow and white maize, pigmented maize remains largely underutilised in the food industry and domestic food preparation. Among consumers, yellow and white maize varieties are the most popular. However, in recent years, coloured varieties have received significant interest from researchers, breeders, and industrialists due to their health-enhancing attributes associated with bioactive compounds. Consequently, a high-performance blue maize hybrid has been developed in Mexico, combining the high-yielding properties of white/yellow maize with the high antioxidant potential of coloured land races [9]. Pigmented cereals, particularly purple wheat, are gaining global popularity as consumption patterns shift towards preventive and nutritive diets [10,11].

Flour from pigmented maize was studied as an ingredient in several traditional and contemporary foods like tortillas, tortilla chips, wholemeal maize bread, mixed wheat/maize bread, pasta, cookies, muffins, snacks, popcorn, extruded breakfast cereals and porridge [12,13,14,15,16,17,18,19,20,21,22,23]. The studies [12,13,14,15,16,17,18,19,20,21,22,23] agreed that pigmented maize conveyed appreciable amounts of anthocyanins and antioxidative activity to the final products; blue-coloured maize was observed as the best source of functional compounds. Moreover, it was revealed that processing methods significantly affected the content of phytochemicals, and most studies suggested that by optimising processing conditions and ingredients, it would be possible to maximise the retention of anthocyanins in maize-based products.

Formulation and processing conditions may severely affect the fate of phytochemicals and, as a consequence, the antioxidant capacity of food formulated from pigmented maize. The antioxidant capacity of the final product is hard to predict as it depends on the structure of antioxidant compounds, the complexity of the food matrix, and the presence of other antagonistic and synergistic compounds [24]. Anthocyanins are chemically unstable and sensitive to hydration, pH changes, metal ions, temperature, oxygen, and light exposure [25,26]. Time of heating exhibited a higher effect on anthocyanin degradation than the temperature in bakery products [27]. Some studies inferred that in bakery goods, anthocyanins are stabilised by interactions with flour proteins and polysaccharides, resulting in high retention percentages (>95%) in buns and biscuits enriched with red grape extract. In food matrices like cookies, anthocyanin retention was significantly improved by modulating pH value using citric acid as an acidulant [16,22]. Rodríguez et al. [18] found that a traditional breadmaking process for yeasted wholemeal maize bread production did not significantly alter the content of anthocyanins and carotenoids in comparison to starting maize flour for white and yellow maize populations but decreased anthocyanins in bread from black maize. The antioxidant capacity of pigment extracts from bread was decreased by at least 50% in comparison to flour, but the authors attributed this to the dilution effect, which was a consequence of the presence of ingredients other than flour in bread formulation. Nevertheless, it is often hard to distinguish between the effect of processing and food matrix on anthocyanin degradation.

In the present study, pigmented maize flours from two Serbian maize genotypes (red and black-coloured) were used in the preparation of a traditional maize-based baked dish called “proja”. Proja is a kind of flat maize bread common in the Balkans with a dense crumb texture similar to cake texture rather than that of wheat bread. Proja based on three ingredients (maize flour, salt, and water) was a staple food for peasants instead of wheat bread. The old-fashioned proja formulation has been modernised in the meantime, and today, common ingredients that accompany maize flour and salt are wheat flour, vegetable oil, eggs, yoghurt, and cheese [28]. It is very popular as a breakfast food, side dish, and appetiser. The objective of this study was to investigate the effect of red and black-coloured maize flour on the content and retention of phenolic compounds and antioxidant activity after baking and the colour and texture properties of proja formulations prepared with different acidifying ingredients (traditional yoghurt/cheese and alternative wheat/rye sourdoughs).

## 2. Materials and Methods

### 2.1. Material Procurement

In this study, two open-pollinated maize varieties, red and black pigmented, originating from the collection within the maize gene bank of the National Institute of Field and Vegetable Crops in Novi Sad, Serbia were used. The maize hybrids were grown and harvested in 2021. Crops were managed under standard agronomic practices. After harvest, kernels were threshed and ground in a hammer mill to obtain wholegrain flour. After homogenisation, the samples were packed in paper bags and stored at ambient temperature in the dark in a storage room.

Other proja ingredients were procured from a local supermarket. Sourdoughs were acquired from two manufacturers: (1) PIP Food Group, Čenej, Serbia (dry durum wheat sourdough (PIP-1); (2) IREKS GmbH, Kulmbach, Germany (dried wheat sourdough (IREX-1), concentrated liquid rye sourdough with fermentative produced lactic and acetic acid (IREX-2 and IREX-3).

### 2.2. Proja Batter Preparation and Baking

Several proja formulations were tested in the study: control (based only on flour, water, baking powder, and salt), two traditional (T1, T2) acidified with cheese or yoghurt and four that contained various forms/levels of sourdough (powder or liquid) as acidifying agents. Ingredients for proja formulations are listed in Table 1. The basic recipe for the preparation of proja batter included thorough homogenisation of salt and baking powder with liquid ingredients (water or yoghurt). Maize flour was added to the liquid ingredient and vigorously mixed to obtain a smooth batter without lumps. If required by the formulation, cottage cheese was mixed in a smooth batter. Batter (80 g) was poured into oiled cups of a non-stick muffin pan with 12 cups and baked at 200 °C for 25 min. After baking and cooling at ambient temperature for at least an hour, proja pieces were removed from the cups, packed into PET boxes and stored at room temperature until further analysis for 24 h.

### 2.3. Determination of Moisture and Total Titratable Acidity

Total titratable acidity (TTA) was determined by crushing 10 g of sample and dispersing it in 100 mL of distilled water. The suspension was homogenised and left to stand for 60 min. Then, 3–5 drops of phenolphtalein are added to the suspension and directly titrated with 0.1 MNaOH until there is an appearance of red colour.

Moisture was determined according to standard AACC method (44–15.02) (AACC Approved Methods of Analysis, 11th Edition).

### 2.4. Determination of Total Phenolics, Flavonoids and Anthocyanins in Maize Flour, Proja Batter and Baked Proja

Phenolic compounds were extracted from ground and sieved samples (flour and dough) using 80% methanol. The ratio of tested material to solvent was 1:10 (*m*/*v*). Extraction was performed in an ultrasonic bath for 45 min. After the extraction, the extracts were centrifuged (10 min, 4000 rpm), and the supernatants were used in further analyses: the content of total phenolics, flavonoids, anthocyanins, as well as the antioxidant capacity of the extract (^•^OH, ∙O_2_^−^, and DPPH-tests). All measurements were determined spectrophotometrically (Perkin Elmer, UV–VIS Lambda Bio 20). Moisture content was determined gravimetrically by drying at 105 °C (3 h) and expressed in %.

The content of total phenolics in methanol extracts was determined based on the reaction of polyphenolic compounds with the Folin-Ciocalteu reagent (partially modified method by Makkar et al. [29]). A dilution series of gallic acid was used to construct the calibration curve. The content of total phenolics is expressed as mg of gallic acid equivalents per g of dry material (mg/g d.m.).

The total flavonoid content in methanol extracts of samples was determined according to the partially modified method of Pękal and Pyrzynska [30]. To the methanol extract (0.5 mL), 1 mL of AlCl_3_ solution in sodium acetate was added, and distilled water was added to the blank sample. After centrifugation (10 min, 4000 rpm), the absorbance was read at 440 nm. The content of flavonoids is expressed as mg of rutin equivalents per g of dry material (mg/g d.m.).

Determination of the total anthocyanin content was performed by the pH differential method [31]. The content of total anthocyanins is calculated as mg of cyanidin-3-glucoside equivalents per g of air-dry weight of material (mg/g d.m.).

Retention of antioxidative phytochemicals was calculated as follows:

$$Retention\left(\%\right)=\frac{{CC}_{after\;baking}}{{CC}_{before\;baking}}\times 100$$

CC_after baking_-compound content (dry matter, d.m.) in sample after baking; CC_before baking_-compound content (d.m.) in batter.

### 2.5. Determination of Antioxidant Capacity of Proja

Superoxide anion inhibition test was applied to test the superoxide anion (∙O_2_^−^) scavenging activity, based on the principle of the ability of the tested extract to inhibit the photochemical reduction of nitro blue tetrazolium chloride (NBT) with superoxide anion [32,33] and expressed as % of inhibition. The reaction medium consisted of 75 μM NBT, 13 mM L-methionine, 0.1 mM EDTA, 13 μM riboflavin, 50 mM KH_2_PO_4_, pH = 7.8 (0.5 mL each) and 0.2 mL of fresh plant material extract (H_2_O in the blank). Test tubes with reaction mixtures were exposed for 15 min to a 20 W LED lamp, and then, after 15 min of resting in the dark, absorbances were read at 560 nm.

Hydroxyl radical (^•^OH) scavenging activity of extracts was assayed by modified method of Li [34] in hydroxyl radical scavenging test (OH-test). The reaction mixture contained 2.7 mL KH_2_PO_4_ buffer (0.1 M, pH 7), 0.1 mL 2-deoxyribose (50 mM), 0.1 mL ferric chloride (10 mM), 0.1 mL H_2_O_2_ (0.015%) and extract solution (0.1 mL). Correction tubes contained phosphate buffer instead of 2-deoxyribose, while control tubes contained extraction solvent instead of sample. After incubation at 37 °C, 60 min, 0.2 mL 0.1 M EDTA and 2 mL TBA reagent solution (20% TCA and 0.5% TBA) were added to all tubes. After boiling for 15 min in a water bath and cooling, the absorbance was measured at 532 nm. The scavenging activity of the hydroxyl radical) was expressed as % of inhibition.

The non-enzymatic activity of the tested samples was determined according to Panda [33], based on the difference in the activity of removing 1,1-diphenyl-2-picrylhydrazyl-radical (DPPH^•^) radical between blank and working samples. 0.3 mL of DPPH methanolic solution and 2.5 mL of methanol were added to the methanol extracts (0.2 mL), while 0.2 mL of methanol was added to the blank test instead of the sample. After 30 min, the absorbance was read at 517 nm. The DPPH-radical scavenging activity is expressed as % in relation to the blank test to which no sample was added.

Retention of antioxidant capacity was calculated according to formula given in Section 2.4.

### 2.6. Colour Analysis

Proja crumb colour (L*, a*, b*) was determined using a Chroma Meter Konica Minolta CR-400 (Konica Minolta Inc., Tokyo, Japan) with a CR-A33 attachment calibrated with a white standard plate CR-A43 D65 illumination and 10° standard observer angle. Measurements were performed on internal crumb surface at cross-section of proja, in triplicate for each proja formulation. From each individual sample, four readings were taken on internal crumb surface. L* value (0–100) measures lightness ranging from black to white; a* (+/−) value measures red/green; b* (+/−) value measures yellow/blue. The whiteness index (WI) is a measure of how closely a surface matches the properties of a perfect reflecting diffuser (ideal white). It is calculated according to formula WI = [(100 – L*)^2^ + (a*)^2^ + (b*)^2^]^0.5^. The degree of total colour difference (ΔE*) between the sample and the control sample is calculated as ΔE* = (ΔL*^2^ + Δa*^2^ + Δb*^2^)^0.5^.

### 2.7. Texture Analysis

Texture analysis was performed on a texture analyser TA-XTplus (Stable Micro Systems, Godalming, UK). Crumb texture was measured using a 36-mm cylindring probe in a compression mode at a test and post-test speed of 1.7 mm/s and 40% strain. Firmness and resilience were extracted from the force–time curves. The peak force of the curve measured during compression was an indicator of crumb hardness. Crumb resilience was calculated as the ratio of the area under the curve during the withdrawal of the first compression to the area under the first compression curve, reflecting the crumb’s capacity to regain its original shape. Measurements were performed 2 h after baking on the lower part of the proja sample obtained after cutting the sample horizontally at the height of the mold.

### 2.8. Statistical Analysis

Data are presented as mean ± standard deviation. All analyses were performed using 2 replications (baking trials) and 3 repetitions (3 samples from each trial). Samples were analysed in triplicates unless otherwise noted. Mean separation was conducted by two-way analysis of variance and Tukey’s (HSD) test at *p* ≤ 0.05. When comparing batter and baked proja, differences between means were determined using paired independent test at *p* ≤ 0.05. Pearson correlation coefficient was applied in correlation analysis (*p* ≤ 0.05). Statistical software TIBCO^®^ Data Science/STATISTICA™14.0.0.15, StatSoft, Hamburg, Germany was applied in data analysis.

The dataset was factor analysed using the principal component and classification (PCA) algorithm of the same software. The dataset was centred to zero mean and scaled to unit variance before calculations.

## 3. Results and Discussion

### 3.1. Moisture Content and Acidity Degree of Proja Crumb

Table 2 presents the moisture content and acidity degree of the crumb of baked proja. Crumb moisture ranged from 35.44% to 49.75%. The highest moisture content was recorded in the T1 proja formulation. TTA spanned in the range 1.6–6.6 mL 1 M NaOH/100 g. The control proja was characterised by lower TTA (1.6, i.e., 2.4 mL 1 M NaOH/100 g for red and black maize) in comparison to other formulations (1.8–2.9 mL 1 M NaOH/100 g for red maize proja and 3.4–6.6 mL 1 M NaOH/100 g but no significant difference was found with exception of IREX-2 black maize proja with the TTA. The crumb acidity (TTA) of all black maize proja was slightly higher than that of red maize proja, but the difference was not statistically significant (*p* > 0.05). Batter pH values (Table 1) were in the range of 5.49–6.20 for red maize and 4.94–6.11 for black maize proja. No significant correlation existed between the batter pH and TTA.

### 3.2. Content of Total Phenolics, Flavonoids and Anthocyanins in Maize Flour, Proja Batter and Baked Proja

Table 3 shows the content of total phenolics, flavonoids, and anthocyanins in maize flours, proja batters and baked proja. Significant differences (*p* < 0.05) in the amounts of these bioactive compounds between black and red maize were found. Black maize flour was a richer source of total phenolics, flavonoids, and anthocyanins than red maize and contained 1.7 times more total phenolics, 10 times more flavonoids, and 1.3 times more total anthocyanins than red maize.

Compared to raw maize flour, phenolic and anthocyanin contents of baked proja were significantly reduced (Table 3). In this respect, batters were initially lower in these compounds due to dilution effects and the presence of other ingredients in proja formulations. Baking further reduced the content of phenolics and anthocyanins, but the differences were not significant in all cases (Table 3). All formulations of proja made from black maize were richer sources of antioxidant compounds than those made from red maize. As seen in Table 3, baked proja made from red maize contained 1.51–1.90 mg GAE/g d.m. total phenolics, 0.05–0.12 mg rutin/g d.m., total flavonoids and 0.02–0.09 mg CGE/g d.m. total anthocyanins versus proja from black maize that contained 2.88–3.49 mg GAE/g d.m. total phenolics, 0.27–0.35 mg rutin/g d.m. total flavonoids and 0.37–0.49 mg CGE/g d.m. total anthocyanins. When different formulations of proja were compared within each maize type, there were no significant differences in the content of total phenolics and flavonoids. Some differences existed in the content of total anthocyanins, especially within black maize proja formulations. However, despite the lack of significant variations in the content of antioxidant compounds within different proja formulations, significant moderate correlations could be observed between baked proja crumb acidity and total phenolics, flavonoids and anthocyanins in baked proja (r^2^ = 0.58/0.62/0.59, respectively). This confirms that, after all, a more acidic environment favours the accessibility of these compounds. When the dataset was correlated separately for each maize type, it was revealed that in black maize proja, total anthocyanins and flavonoids were significantly positively related to crumb acidity degree (r^2^ = 0.52 and r^2^ = 0.54). On the other hand, in the case of red maize proja, only a significant positive correlation between the content of phenolics and crumb acidity (r^2^ = 0.45) could be observed. Different relations between the contents of bioactive compounds and crumb acidities observed separately in red and black maize proja datasets are probably due to the different profiles of these compounds in each maize type.

Previous research showed that an acidic environment increases the level and stability of phenolic and anthocyanin compounds in food matrices. Li et al. [16]) concluded that, among 3 tested acidulants, citric acid produced the lowest pH (dough pH 3.3–4.5 and cookie pH 3.4–4.6) and highest retention of total anthocyanins in blue corn cookies. Žilić et al. [22] confirmed that citric acid (crumb pH range 3.55–4.59) significantly increased the total flavonoids and anthocyanins content in cookies prepared from blue popping maize. The presence of sourdough in the formulation of bread enriched with purple potato and citrus significantly increased the levels of total phenols and bread antiradical activity [35]. The present study also showed significant correlations between the levels of antioxidant compounds and proja crumb acidity, but the range in which the acidity of differently formulated proja varied was relatively narrow (batter pH around 5–6; TTA mostly between 1.5 and 4.2) (Table 1 and Table 2) to elicit more profoundly the differences.

### 3.3. Antioxidant Properties of Baked Proja

**Figure 1:**
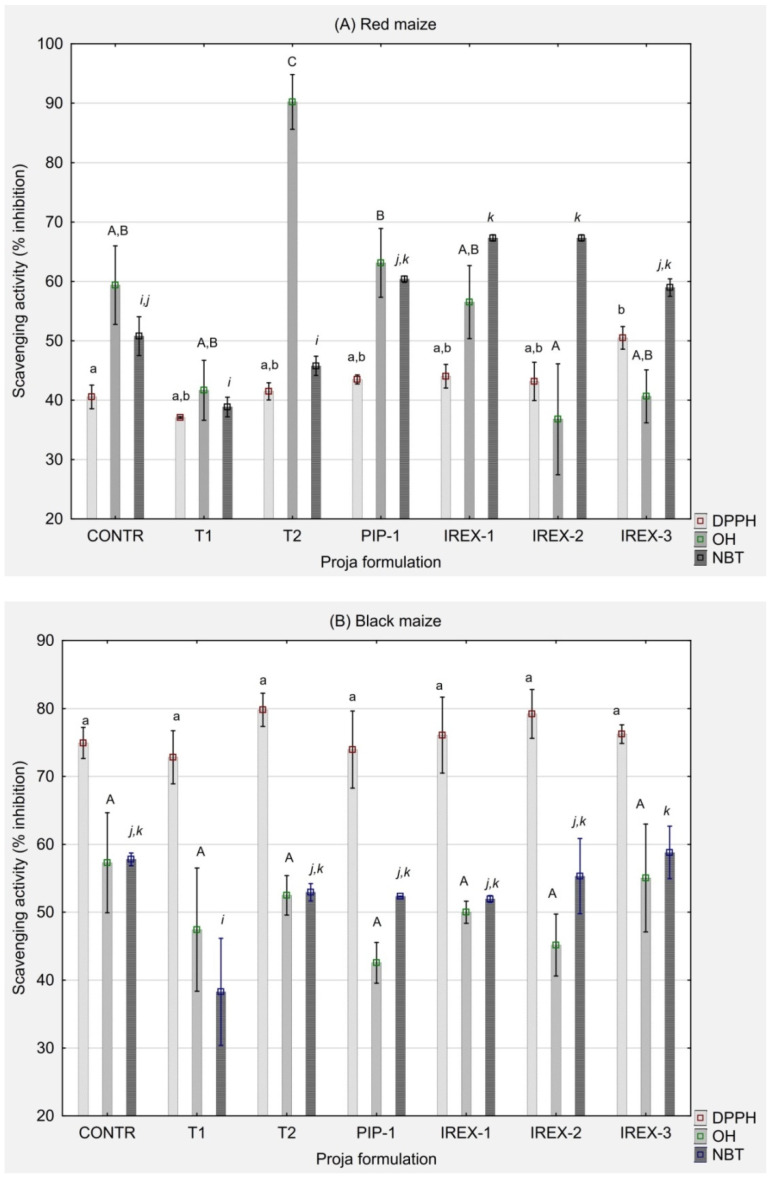
Antioxidant capacity of proja made from red (**A**) and black (**B**) coloured maize. DPPH represents DPPH-radical scavenging activity; OH denotes hydroxyl radical scavenging activity; NBT represents superoxide anion inhibitory activity. The vertical bars represent mean ± standard deviation of each data point (n = 6). Bars designated with equal letters of the same typesetting are not statistically significant (*p* > 0.05) according to Tukey’s (HSD) test.

Antioxidant properties of proja from red and black maize are depicted in Figure 1 and were characterized by the ability to inhibit ∙DPPH free radical, hydroxyl radical ∙OH and superoxide radical ∙O_2_^−^ (NBT dye assay). There were differences in ∙DPPH, ∙OH and ∙O_2_^−^ inhibition between red and black maize proja presumably due to different profiles and content of antioxidant compounds. The results indicated that black maize proja exhibited significantly higher antioxidant activity against ∙DPPH free radicals with the ability to inhibit 73–80% ∙DPPH radicals, in contrast to red maize proja that inhibited 37–54% of ∙DPPH. Significant correlations existed between the inhibitory activity against ∙DPPH radical and the content of antioxidant compounds in pigmented maize: r^2^ = 0.97 for total anthocyanins, r^2^ = 0.96 for total flavonoids, and r^2^ = 0.95 for total phenolics. Activities against hydroxyl and superoxide radicals were similar between red and black maize proja formulations, but no significant correlations existed concerning the content of phenolics, flavonoids and anthocyanins when the complete dataset was correlated. When datasets for red and black maize were separately correlated, total flavonoids of black maize proja were significantly moderately correlated with inhibition of ∙OH and ∙O_2_^−^ (r^2^ = 0.51 and 0.62, respectively). Higher crumb acidity degree could be associated with more pronounced antiradical action against ∙DPPH (r^2^ = −0.65 for crumb TTA). When only data for red maize proja was correlated, the more acidic crumb was found to favour both ∙DPPH and ∙O_2_^−^ inhibition (r^2^ = 0.58, i.e., 0.59, respectively).

### 3.4. Effect of Baking and Acidity of Proja Formulations on the Retention of Antioxidant Compounds and Antioxidant Activity

#### 3.4.1. Retention of Antioxidant Compounds

**Figure 2:**
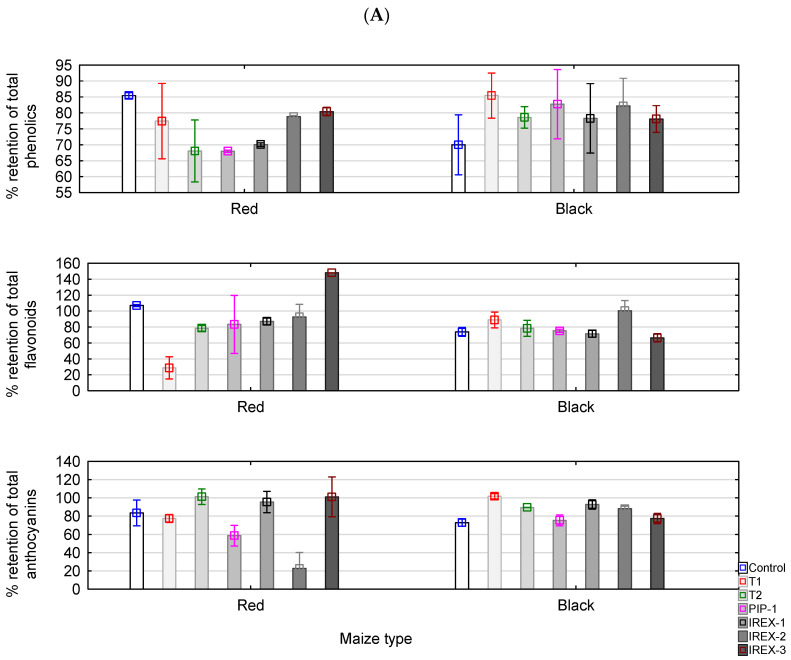

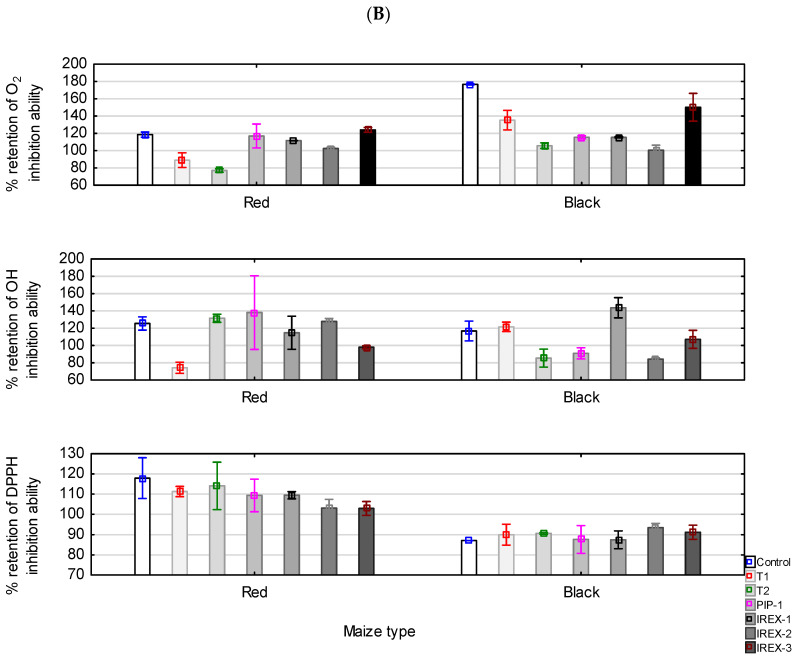
Effect of baking on the retention of antioxidant acitivity (**A**) and phytochemicals (**B**) in proja. The vertical bars represent mean ± standard deviations of each data point (n = 6).

Data on the retention of total phenolics, total flavonoids, and total anthocyanins following baking are displayed in Figure 2. In red maize proja, 67–85% of total phenolics were retained (Figure 2A). Similar ranges could be observed in black maize proja, 69–85%. When different proja formulations were individually compared to their corresponding counterparts between red and black datasets, slightly higher retentions were noted in proja from black maize but did not reach statistical significance. The retention pattern for total flavonoids and anthocyanins was more scattered, with higher variations among the analysed proja formulations. For red maize proja, it ranged from 28% to 137% for total flavonoids and from 22 to 101% for total anthocyanins (Figure 2A). On the other hand, in black maize proja, retention of total flavonoids and anthocyanins was in the range of 67–100%, i.e., 73–102%, respectively (Figure 2A). Observing the whole dataset in general, in the majority of samples, we see that flavonoids were retained in the range of 66–90%, while anthocyanin retention indices were between 58 and 93%. Proja made from black maize showed higher retention of all observed bioactive compounds in all formulations other than control. In contrast, in red maize proja, the control sample had higher retention of total phenolics and flavonoids compared to the majority of other proja formulations except for the retention of total flavonoids in IREX-3 formulations.

Retention of total phenolics, flavonoids and anthocyanins seemed to be enhanced in more acidic formulations of black maize proja as revealed by significant positive correlations with acidity degree at r^2^ = 0.70 (total phenolics), 0.82 (total flavonoids) and 0.47 (total anthocyanins). In red maize proja, however, crumb acidity could not be correlated to the retention of any observed antioxidant compound.

It has been frequently reported that phenolic compounds increase after thermal treatments. The increases can be partially attributed to the release of phenolics from bound forms. In contrast to our findings, Simić et al. [21] found that total phenolics increased after baking in wheat bread enriched with 30% (replacement level) wholegrain blue and dark-red maize flour, emphasising that the changes were more pronounced in the bread crust (30% increase in comparison to raw maize flour). The differences in our results might be due to differences in product type, as proja is not similar to bread. On the other hand, flavonoids are considered less thermally stable compounds due to their conjugated forms. But, this might not be taken as a rule since contradictory results exist in the literature [24] due to yet unrevealed, complex interactions between flavonoid structure, food matrix and processing conditions. Significant reductions in total flavonoids and anthocyanins were reported in mixed wheat-coloured maize breads, especially intensive in the crust [21].

Despite thermal degradation, the remaining amounts of total phenolics, flavonoids and anthocyanins in baked proja from red and black maize were appreciable and may be expected to enhance the functionality and health-promoting potential of the baked product. Proja samples from this study were richer sources of total phenolics and anthocyanins in comparison to tortilla chips from blue corn [20] and muffins from purple maize [23]. Similar amounts of anthocyanins and somewhat less phenolics were reported in red maize tortillas [12,15], red maize chips [15] and bread from mixed wheat–dark red/blue maize [21]. Similar ranges of these compounds were also reported for cookies made from dark-red and blue popping maize and blue standard maize [23].

#### 3.4.2. Retention of Antioxidant Activity in Baked Proja

Baking affected the antioxidant properties of proja. Figure 2B displays the retention of antioxidant properties after baking. As can be observed in the majority of cases, increased antioxidant capacity was noted after baking (% retention above 100%). Increased inhibitory activity against DPPH, hydroxyl and superoxide inhibition were measured for most formulations of red and black maize proja, except DPPH inhibition in black maize proja. Nevertheless, around 90% of DPPH inhibitory activity was retained. Retention of OH inhibition capacity in both maize types was not univocal within formulations and ranged from 70% to 170% (Figure 2B). The results indicate that the phenolic compounds remaining in proja after baking were able to retain their activity. Increased antioxidant activity after baking is a frequent pattern and can be explained to be in part due to the liberation of bound phenolic compounds from the matrix and the presence of other products of thermal degradation that exhibit antioxidant properties. Higher antioxidant capacity after cooking pasta prepared from blue maize was reported by Camelo-Méndez et al. [13]. Increased antioxidant activity in cookies after baking due to the formation of Maillard reaction products has been shown in the study of Žilić et al. [22]. The slight difference in the retention of inhibitory activities against tested free radicals in proja from red and black maize is presumably due to different profiles and content of phenolic compounds in these maize cultivars. Different proja formulations significantly affected the retention of the capacity to inhibit free radicals. In the case of DPPH, statistical significance existed among formulations of proja made from red maize in contrast to black maize proja, where different formulations exhibited similar retentions of antioxidant potential that ranged from 87.1 to 93.52%. There was a weak but significant inverse correlation between acidity degree and retention of DPPH inhibitory activity (r^2^ = −0.35), indicating that higher acidity did not favour the retention of antioxidant activity after proja baking. Considering the retention of activities against superoxide and hydroxyl radicals, variations among different proja formulations were broad but did not follow an explicable trend, and no correlations were found. However, when correlations were performed on separate datasets, the pattern of antioxidant activity retention in red maize proja coincided with the retention of anthocyanins and flavonoids after baking: the remaining inhibitory activities against DPPH could be correlated to the retention of anthocyanins (r^2^ = 0.46), and activities against superoxide were highly correlated to the retention of flavonoids (r^2^ = 0.71) in proja after baking.

### 3.5. Colour Properties of Proja Made from Red and Black Maize

Colour measurements were performed on the samples of crumbs of baked proja and were expressed in the L*a*b* colour space (Table 4). The colour properties of baked proja significantly differed depending on the type of pigmented maize flour. Black maize gave a dark tone to the proja crumb, while red maize shifted the proja crumb colour to a lighter and yellow range. CIE L* values for black maize proja were lower by 150–160% compared to those of red counterparts. CIE a* values ranged from 2.50 to 5.97 and from 4.25 to 6.95 in red and black maize proja, respectively. Crumbs of red maize proja had CIE b* 3.6 to 7.2-fold higher than black maize proja. There were no significant differences among formulations of black maize proja in whiteness index (WI), lightness (L*), and yellow nuances (b*), which may be due to the generally dark colour attributed to the original colour associated with higher concentration and specific profile of anthocyanins in the black maize. But, the formulations differed in CIE a*. In contrast, red maize proja formulations differed significantly from each other in all colour parameters. Lighter proja crumbs were associated with more intense red and yellow nuances, which were exhibited by three red maize formulations: T2, PIP-1, and IREX-2. Similarly, in black maize proja, the highest red tones were also observed in T2 and IREX-2 formulations, including T1. For black maize proja, the total colour difference (ΔE) in comparison to the corresponding control was small and not perceivable by the human eye (ΔE < 3). In contrast, ΔE for red maize proja samples T2 and IREX-2 was distinctively different from the control (ΔE > 3). These samples were lighter and more red in comparison to the corresponding control. A more acidic environment in proja batter and crumb was associated with higher ΔE of baked proja according to significant correlations with pH (r^2^ = −0.70, *p* < 0.05) and TTA (r^2^ = 0.47, *p* < 0.05).

Moderately low pH values in the range 4–5 are required for the stable colour of anthocyanins in the range red-pink-violet. Žilić et al. [22] noted that cookies made from blue corn at pH 3.55 and 4.41 (adjusted with citric acid) appeared pink, i.e., pale pink. Formulations T1, T2, PIP-1 and IREX-2 have lower batter pH (<6.0) in comparison to other formulations (Table 1). When data from all samples were correlated, batter pH was significantly inversely correlated to CIE a* (r^2^ = −0.53) and positively to CIE b* (r^2^ = 0.31), which associate lower pH values with more red and less yellow samples. The TTA of baked proja was strongly positively related to red tones (r^2^ = 0.72) but negatively to CIE L* and CIE b* (r^2^ = −0.43; −0.47, respectively), also showing a tendency that more acidic crumb favours more red, less yellow and dark crumb. On the other hand, when the acidity degree of the baked proja only from red maize was correlated, significant positive correlations to CIE L* CIE b* and CIE a* were found (r^2^ = 0.71, 0.49, 0.58, respectively), indicating that formulations made from red maize with higher crumb acidity were lighter, more yellow, and red in tone. In black maize proja, crumb TTA was also significantly positively correlated with red nuance (r^2^ = 0.65). A significant moderate positive correlation between total anthocyanins (r^2^ = 0.59) to acidity degree in baked proja supports the assumption that an acidulous environment favours anthocyanins stability and retention.

### 3.6. Textural Properties

The textural characteristics of the proja samples are displayed in Table 5. Crumb hardness was significantly affected (*p* < 0.05) by the maize type and proja formulation. Control red maize proja was significantly softer than the black maize proja. The presence of different acidifying ingredients in proja formulation increased crumb hardness. The highest increase was observed in T2 samples, probably due to the presence of cheese pieces. Crumb resilience describes the elastic properties of the crumb. The variation in resilience was due to different proja formulations. The presence of cheese in proja (T2 samples) significantly decreased the crumb elasticity. In comparison to the corresponding controls, the crumb resilience slightly decreased in most proja formulations without reaching statistical significance. Crumb resilience was significantly positively correlated with batter pH and inversely correlated to TTA, indicating that a more acidic environment lowers the elastic properties of the crumb.

### 3.7. Principal Component Analysis (PCA)

**Figure 3:**
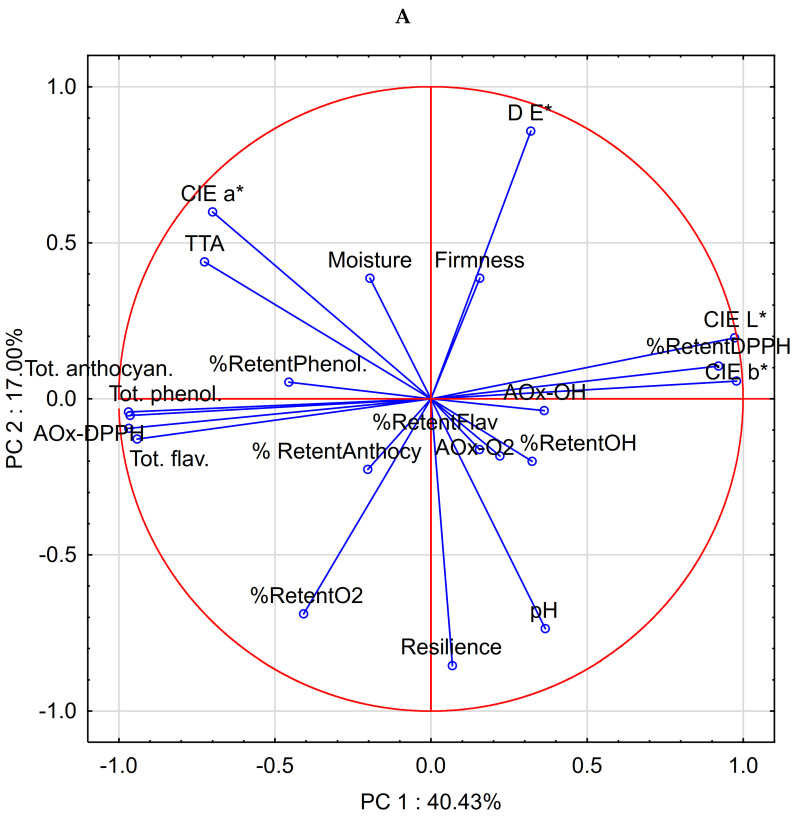

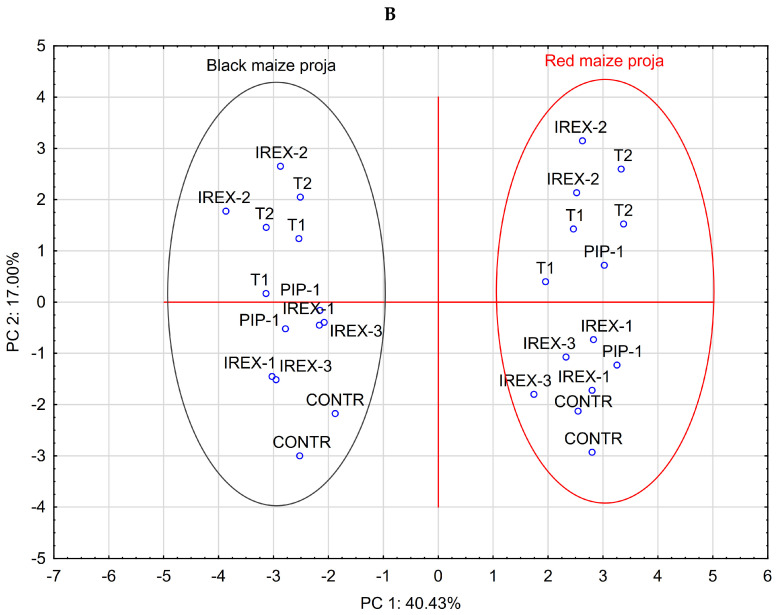
PCA loading (**A**) and score (**B**) plots of proja properties (PC1 versus PC2).

PCA was performed on the dataset to explain the variability in the data. The analysis yielded four PCs, which explains 78.8% of the total variance in the data. Variable contributions are presented in Table 6 to express the correlation between old variables and newly defined principal components (PCs). The first principal component PC1 explained 40.4% variability of the data and correlated well with colour properties (L*, a*, b*), content of bioactive compounds (phenolics, flavonoids, anthocyanins), crumb acidity (TTA), antioxidant activity and its retention (DPPH). The majority of the variables were negatively correlated to PC1 due to inverse correlations between many of the original variables. Proja with higher crumb acidity (TTA) tends to be lighter in colour, more reddish, less yellow, and higher in the content of bioactive compounds and has higher antioxidant activity against DPPH but exerts lower retention of inhibitory activity against DPPH after baking. The second component correlated with batter pH, crumb resilience, retention of ∙O2-antiradical activity, and total colour difference. More acidic proja batter (lower pH) can be associated with less elastic crumb (lower crumb resilience), higher total colour difference in comparison to the corresponding control and lower retention of inhibitory activity against ∙O2- radical. The third component correlated negatively with crumb moisture, while the fourth factor was associated with the antioxidant activity against ∙O2-radical. Figure 3 shows the loadings (A) and scores (B) of the first two PCs against each other. According to PC1 and PC2, the proja samples are clearly separated between the red and black maize. Black maize proja samples are distinct from the red maize samples due to their high content of bioactive compounds and DPPH antioxidant capacity, while red maize proja differs in colour properties (lightness and yellowness) and retention of DPPH antioxidant activity. Figure 3B shows that there was a pattern in similarities between different proja formulations. The formulations based on sourdoughs IREX-1 and IREX-3 were located in the lower left and right quadrants of the PCA plot and were closer to the control samples, indicating higher similarities with the corresponding controls. The traditional formulations based on yoghurt and cheese (T1, T2) and IREX-2 sourdough were grouped on the opposite side of the PC 1 factor plane showing mutual similarities. Different proja formulations were discriminated along the PC2 loading axis according to the total colour difference, crumb resilience, batter pH and retention of ∙O2- inhibitory activity. Proja samples made with sourdoughs IREX-1 and IREX-3 exerted similarities by having lower total colour difference and lower crumb resilience in comparison to T1, T2, and IREX-2.

In the bakery industry, the major problem in processing anthocyanin-rich products is the thermal degradation of anthocyanins, which extent depends on many factors, including the initial level of anthocyanins and characteristics of the food matrix (presence of sucrose, Maillard reaction products, acidifying agents, etc.). The stability and content of anthocyanins can be increased by increasing the acidity. The performance of the ingredients used in this study as acidifying ingredients (traditional (yoghurt and cheese) and alternative (dry wheat sourdough PIP-1 and IREX-1 and concentrated liquid rye sourdough (IREX-2 and IREX-3)) thus can be viewed from this aspect. As discussed in Section 3.1, crumb acidity (TTA) increased in comparison to the control proja but did not significantly vary among the samples with different acidifying ingredients. After baking, a similar trend was observed: the analysed bioactive compounds showed little variations amongst the samples with different acidifying ingredients within the same maize type (see Section 3.2). In red maize proja, the highest content of anthocyanins was observed in the samples with cheese (T2) and IREX-3 sourdough. In black maize, the proja samples with cheese (T2), IREX-1, and IREX-2 had the highest anthocyanins. Anthocyanin retention was the highest in red maize proja with T2, IREX-1 and IREX-3, while in black maize proja, the proja with yoghurt showed the highest retention, followed by IREX-1, IREX-2 and T2. All studied acidifying ingredients provided stable and satisfying colours to the products (see Section 3.5).

## 4. Conclusions

This study demonstrated the performance of the flour of two pigmented maize genotypes (red and black) in the preparation of the traditional Serbian maize baked dish “proja”. During proja baking, 15–33% of the total phenolics were degraded in both red and black-coloured maize, while the degradation levels for total flavonoids and anthocyanins varied over a wider range among the samples, from 70% degradation to liberation (>100% retention). Much of the antioxidant activity in baked proja was retained: 90% of activity against DPPH was retained in black-maize proja, while in red maize proja, baking increased the antioxidant activity. At the same time, inhibition of superoxide and hydroxyl radicals was similar in the red and black maize proja. The inhibitory activity against DPPH radicals was highly correlated with the content of total phenolics, flavonoids, and anthocyanins in the red and black maize proja. The more acidic crumb of different proja formulations favoured the amount and retention of antioxidant compounds and inhibitory activities against the tested free radicals. It could also be associated with increasing lightness and red/yellow tones of the crumb, particularly in the red maize proja. Crumb hardness in both red and black maize proja significantly increased with added ingredients compared to the corresponding controls. Our results showed that red and black maize flour can be used as sources of appreciable amounts of antioxidant compounds and may be expected to enhance the functional properties of this simple and affordable dish. The findings can be applied in practice, may inspire food manufacturers innovating traditional foods using non-traditional ingredients, and increase the valorisation potential in novel industrial applications.

## Figures and Tables

**Table 1:** Proja dough formulations.

Ingredients (g)	Proja Formulations						
	Control	T1	T2	PIP-1	IREX-1	IREX-2	IREX-3
Maize flour	100	100	100	100	100	100	100
Baking powder	2.5	2.5	2.5	2.5	2.5	2.5	2.5
Salt	1.8	1.8	1.8	1.8	1.8	1.8	1.8
Water	100	50	-	-	-	-	-
Cottage cheese	-	100	-	-	-	-	-
Yoghurt	-	-	100	-	-	-	-
Dry wheat sourdough: PIP IREX	- -	- -	- -	10 -	- 10	- -	- -
Liquid wheat sourdough: IREX	-	-	-	-	-	7	4
Batter pH red maize black maize	6.20 ± 0.27 ^c^ 6.11 ± 0.30 ^b,c^	5.54 ± 0.28 ^a–c^ 4.94 ± 0.18 ^a^	5.72 ± 0.19 ^b,c^ 5.96 ± 0.22 ^b,c^	5.89 ± 0.19 ^b,c^ 5.84 ± 0.09 ^b,c^	6.11 ± 0.25 ^b,c^ 6.09 ± 0.30 ^b,c^	5.49 ± 0.22 ^a,b^ 4.98 ± 0.28 ^a^	6.13 ± 0.20 ^b,c^ 5.71 ± 0.09 ^b,c^

Values reported are means ± the standard deviation for baked proja (n = 6). ^a–c^ Values within raws with different superscript letters are significantly (*p* < 0.05) different between all samples of baked proja using two-way ANOVA (HSD test).

**Table 2:** Moisture content (%) and total titratable acidity (TTA) (ml 1 M NaOH/100 g) in crumb of baked proja made from red and black maize.

Parameter	Proja Formulations						
	Control	T1	T2	PIP-1	IREX-1	IREX-2	IREX-3
**Red maize**							
Moisture content	37.7 ± 3.9 ^a,b^	49.7 ± 5.2 ^c^	42.8 ± 4.4 ^a–c^	44.3 ± 4.6 ^a–c^	42.7 ± 4.4 ^a–c^	40.9 ± 4.2 ^a–c^	35.4 ± 3.7 ^a^
TTA	1.6 ± 0.4 ^a^	2.1 ± 0.6 ^a^	1.8 ± 0.47 ^a^	3.1 ± 0.8 ^a^	2.3 ± 0.6 ^a^	3.6 ± 0.9 ^a^	2.9 ± 0.8 ^a^
**Black maize**							
Moisture	42.9 ± 4.4 ^a–c^	47.6 ± 2.5 ^b,c^	38.5 ± 4.0 ^a–c^	42.1 ± 4.4 ^a–c^	41.0 ± 4.2 ^a–c^	46.0 ± 2.4 ^a–c^	44.3 ± 2.3 ^a–c^
TTA	2.4 ± 0.6 ^a^	4.1 ± 1.1 ^a,b^	4.2 ± 1.1 ^a,b^	3.4 ± 0.9 ^a^	3.8 ± 1.0 ^a^	6.6 ± 1.7 ^b^	4.03 ± 1.1 ^a,b^

Values reported are means ± the standard deviation for baked proja (n = 6). ^a–c^ Values within rows with different superscript letters are significantly (*p* < 0.05) different between the samples of baked proja from red and black maize using two-way ANOVA (HSD test).

**Table 3:** Content of total phenolics, flavonoids and anthocyanins in proja dough and baked proja.

Maize	Sample/Product		Total Phenolics (mg GAE/g d.m.)	Total Flavonoids (mg rutin/g d.m.)	Total Anthocyanins (mg CGE/g d.m.)
**Red**	Raw flour		2.59 ± 0.13 ^b^	0.05 ± 0.00 ^a^	0.09 ± 0.02 ^a,b^
	Control	batter	1.95 ± 0.11	0.11 ± 0.01	0.04 ± 0.07
		baked	1.67 ± 0.11 ^a^	0.12 ± 0.01 ^a^	0.04 ± 0.01 ^a,b^
	T1	batter	2.46 ± 0.12	0.09 ± 0.01 *	0.10 ± 0.01
		baked	1.90 ± 0.20 ^a^	0.03 ± 0.01 ^a^	0.07 ± 0.01 ^a,b^
	T2	batter	2.61 ± 0.11 *	0.13 ± 0.00 *	0.10 ± 0.00
		baked	1.77 ± 0.18 ^a^	0.10 ± 0.00 ^a^	0.10 ± 0.01 ^b^
	PIP-1	batter	2.48 ± 0.23	0.07 ± 0.03	0.07 ± 0.00 *
		baked	1.69 ± 0.15 ^a^	0.05 ± 0.00 ^a^	0.04 ± 0.01 ^a,b^
	IREX-1	batter	2.16 ± 0.04 *	0.12 ± 0.02	0.06 ± 0.00
		baked	1.51 ± 0.04 ^a^	0.10 ± 0.01 ^a^	0.06 ± 0.01 ^a,b^
	IREX-2	batter	2.24 ± 0.08 *	0.12 ± 0.02	0.09 ± 0.00 *
		baked	1.76 ± 0.07 ^a^	0.10 ± 0.00 ^a^	0.02 ± 0.01 ^a^
	IREX-3	batter	2.28 ± 0.22	0.07 ± 0.02	0.09 ± 0.00
		baked	1.83 ± 0.21 ^a^	0.09 ± 0.04 ^a^	0.09 ± 0.02 ^b^
**Black**	Raw flour		4.34 ± 0.25 ^d^	0.50 ± 0.06 ^c^	0.70 ± 0.03 ^f^
	Control	batter	4.15 ± 0.53	0.45 ± 0.05	0.51 ± 0.02 *
		baked	2.88 ± 0.02 ^b,c^	0.33 ± 0.06 ^b^	0.37 ± 0.00 ^c^
	T1	batter	3.64 ± 0.29	0.30 ± 0.02	0.38 ± 0.00
		baked	3.10 ± 0.01 ^b,c^	0.27 ± 0.05 ^b^	0.38 ± 0.01 ^c^
	T2	batter	4.22 ± 0.14 *	0.38 ± 0.00	0.53 ± 0.01 *
		baked	3.31 ± 0.04 ^c^	0.30 ± 0.04 ^b^	0.47 ± 0.01 ^e^
	PIP-1	batter	3.94 ± 0.55	0.42 ± 0.01 *	0.53 ± 0.01 *
		baked	3.23 ± 0.03 ^c^	0.32 ± 0.00 ^b^	0.40 ± 0.03 ^c,d^
	IREX-1	batter	4.48 ± 0.25	0.44 ± 0.01 *	0.53 ± 0.03
		baked	3.49 ± 0.29 ^c^	0.32 ± 0.02 ^b^	0.49 ± 0.00 ^e^
	IREX-2	batter	3.63 ± 0.28	0.34 ± 0.00	0.52 ± 0.01
		baked	2.98 ± 0.09 ^b,c^	0.35 ± 0.04 ^b^	0.46 ± 0.02 ^d,e^
	IREX-3	batter	4.09 ± 0.06 *	0.44 ± 0.05	0.52 ± 0.01 *
		baked	3.20 ± 0.22 ^b,c^	0.29 ± 0.05 ^b^	0.40 ± 0.02 ^c,d^

Values reported are means ± the standard deviation for baked proja (n = 6). ^a–f^ Values within a column with different superscript letters designate significant differences (*p* < 0.05) between the means of flour and baked proja using two-way ANOVA (HSD test). * Indicates significant statistical difference (*p* < 0.05) between paired groups (batter and baked proja) using paired independent *t*-test.

**Table 4:** Colour properties in L*a*b* colour space of the top surface of baked proja made from pigmented maize.

Proja Formulation	Colour Parameters				
	L*	a*	b*	WI	ΔE*
**Red maize**					
Control	35.20 ± 1.14 ^b^	2.93 ± 0.51 ^a–c^	9.29 ± 0.96 ^d,e^	34.46 ± 1.03 ^b^	^-^
T1	35.55 ± 1.80 ^b^	3.25 ± 0.43 ^c^	7.04 ± 0.70 ^c^	35.08 ± 1.73 ^b^	2.87 ± 0.75 ^c–e^
T2	38.81 ± 1.80 ^c^	4.05 ± 0.30 ^d^	8.89 ± 0.93 ^d,e^	38.02 ± 1.67 ^c^	4.09 ± 1.27 ^f^
PIP-1	38.62 ± 1.14 ^c^	3.14 ± 0.30 ^b,c^	8.86 ± 0.92 ^d,e^	37.90 ± 1.01 ^c^	3.63 ± 0.92 ^e,f^
IREX-1	36.38 ± 1.82 ^b^	2.60 ± 0.41 ^a,b^	8.27 ± 1.03 ^d^	35.78 ± 1.69 ^c^	2.41 ± 0.91 ^a–d^
IREX-2	40.03 ± 1.60 ^c^	5.97 ± 0.37 ^f^	9.72 ± 1.08 ^e^	38.95 ± 1.44 ^c^	5.90 ± 1.28 ^g^
IREX-3	35.75 ± 1.86 ^b^	2.48 ± 0.31 ^a^	8.31 ± 0.91 ^d^	35.15 ± 1.73 ^b^	2.21 ± 0.75 ^a–d^
**Black maize**					
Control	23.92 ± 0.86 ^a^	4.49 ± 0.30 ^d,e^	2.68 ± 0.35 ^b^	23.74 ± 0.84 ^a^	^-^
T1	24.69 ± 0.83 ^a^	6.59 ± 0.46 ^g^	1.72 ± 0.50 ^a,b^	24.38 ± 0.79 ^a^	2.59 ± 0.51 ^b–e^
T2	24.30 ± 0.84 ^a^	6.84 ± 0.29 ^g^	0.98 ± 0.45 ^a^	23.86 ± 1.09 ^a^	3.07 ± 0.21 ^d–f^
PIP-1	24.18 ± 0.70 ^a^	4.68 ± 0.42 ^e^	1.10 ± 0.52 ^a^	24.03 ± 0.68 ^a^	1.79 ± 0.50 ^a–c^
IREX-1	23.89 ± 0.74 ^a^	4.25 ± 0.21 ^d,e^	1.37 ± 0.34 ^a^	23.76 ± 0.72 ^a^	1.49 ± 0.43 ^a,b^
IREX-2	23.65 ± 0.83 ^a^	6.95 ± 0.42 ^g^	1.45 ± 0.43 ^a^	23.31 ± 0.79 ^a^	2.91 ± 0.30 ^c–e^
IREX-3	23.44 ± 0.68 ^a^	4.40 ± 0.25 ^d,e^	1.60 ± 0.45 ^a,b^	23.30 ± 0.66 ^a^	1.37 ± 0.46 ^a^

^a–g^ Values within a column with different superscript letters are significantly (*p* < 0.05) different.

**Table 5:** Texture properties of proja made from pigmeneted maize.

Proja Formulation	Crumb Texture Properties	
	Hardness (g)	Resilience (%)
**Red maize**		
Control	2053.48 ± 251.63 ^a^	25.42 ± 1.13 ^b–d^
T1	2110.63 ± 323.25 ^a^	23.19 ± 1.41 ^b,c^
T2	9161.56 ± 431.71 ^g^	16.90 ± 0.99 ^a^
PIP-1	4551.29 ± 169.15 ^c,d^	24.62 ± 0.97 ^b–d^
IREX-1	3446.88 ± 51.42 ^b^	24.91 ± 0.33 ^b–d^
IREX-2	4951.63 ± 42.91 ^d,e^	17.16 ± 0.99 ^a^
IREX-3	5763.36 ± 15.10 ^e,f^	25.63 ± 1.62 ^b–d^
**Black maize**		
Control	3713.54 ± 115.34 ^b,c^	27.18 ± 0.84 ^d^
T1	3969.13 ± 236.24 ^b,c^	23.03 ± 1.39 ^b^
T2	6173.92 ± 348.57 ^f^	15.78 ± 1.21 ^a^
PIP-1	5109.18 ± 537.74 ^d,e^	26.57 ± 2.15 ^c,d^
IREX-1	3383.20 ± 273.46 ^b^	23.25 ± 0.25 ^b,c^
IREX-2	3749.82 ± 334.43 ^b,c^	17.12 ± 0.73 ^a^
IREX-3	3489.25 ± 340.18 ^b^	23.94 ± 0.62 ^b–d^

^a–g^ Values within a column with different superscript letters are significantly (*p* < 0.05) different.

**Table 6:** Results of PCA in maize proja samples: factor loadings.

Variable	PC 1	PC 2	PC 3	PC 4
Moisture	−0.195488	0.386069	**−0.757916**	−0.124298
CIE L*	**0.972581**	0.194805	0.038914	0.100618
CIE a*	**−0.700153**	0.598311	0.180155	0.046076
CIE b*	**0.979018**	0.056842	0.050564	0.139616
D E*	0.320088	**0.857348**	0.223341	0.090444
Tot. anthocyan.	**−0.968970**	−0.042776	0.081670	−0.195823
Tot. phenol.	**−0.963911**	−0.053878	0.039666	−0.145032
Tot. flav.	**−0.941568**	−0.128764	0.188236	−0.115592
pH	0.366531	**−0.736491**	0.300076	−0.135616
TTA	**−0.725543**	0.438184	0.207404	0.264336
%RetentPhenol.	−0.455403	0.053145	−0.121501	0.524467
%RetentFlav	0.155140	−0.162705	0.659243	0.319850
% RetentAnthocy	−0.202143	−0.226559	−0.103077	−0.528551
AOx-DPPH	**−0.968464**	−0.094679	0.198341	−0.086478
AOx-OH	0.362708	−0.038254	0.126948	**−0.855088**
AOx-O2	0.220316	−0.183760	0.637970	0.396418
%RetentDPPH	**0.921358**	0.104600	−0.178629	−0.023985
%RetentOH	0.324338	−0.200808	0.301355	−0.252500
%RetentO2	−0.408402	**−0.689490**	0.054466	0.133201
Firmness	0.155954	0.386127	0.587324	−0.539277
Resilience	0.068670	**−0.854084**	−0.309196	0.167662
Proportion of explained variance (%)	40.43	17.00	11.08	10.33

Loading values > 0.7000 are marked in boldface type as significant.

## Data Availability

The original contributions presented in the study are included in the article, further inquiries can be directed to the corresponding author.
